# Supplementary material for: Content-rich biological network constructed by mining PubMed abstracts
Source: BMC Bioinformatics. 2004 Oct 8;5:147. doi: 10.1186/1471-2105-5-147 (PMC528731; doi:10.1186/1471-2105-5-147)
Supplement: Additional File 2 — The original results of the above study (non-essential files are deleted to keep the file size under the limit set by BMC bioinformatics). [file 1471-2105-5-147-S2.bz2 › chilibotAdditionalFile2/dip20/712ID9732274E18049/html/PNP_RNE.html]

 


 **PNP** and **RNE** 
  
Found 60 abstracts in PubMed, retrieved 20.  
 

 What does Google say? 
 PDF only 
| .edu only 

---

**Interactive relationship** (e.g. stimulation, inhibition, etc)

**Inhibitory relationship**- We show that purified CspE impedes poly A mediated 3 to 5 exonucleolytic decay by PNPase  [ **PNP** ]  by interfering with its digestion through the poly A tail and also inhibits both internal cleavage and poly A tail removal by RNase E  [ **RNE** ] .  Ref: 11390393 J Biol Chem, 2001
- RNase E  [ **RNE** ]  initiates the decay of Escherichia coli RNAs by cutting them internally near their 5 end and is a component of the RNA degradosome complex, which also contains the 3 exonuclease PNPASE  [ **PNP** ]  Recently, RNase E  [ **RNE** ]  has been shown to be able to remove poly A tails by what has been described as an exonucleolytic process that can be blocked by the presence of a phosphate group on the 3 end of the RNA.  Ref: 11328869 Nucleic Acids Res, 2001
**Neutral relationship**- A Streptomyces coelicolor functional orthologue of Escherichia coli RNase E  [ **RNE** ]  shows shuffling of catalytic and PNPase  [ **PNP** ]  binding domains.  Ref: 12675796 Mol Microbiol, 2003
- PNPase  [ **PNP** ]  is not essential, as RNase E  [ **RNE** ]  remains fully active towards mRNAs in  **rne**   **pnp**  mutants.  Ref: 10411735 Mol Microbiol, 1999
- The ribosomal protein S1, which is known to interact with sequences at the 5 ends of mRNA molecules during the initiation of translation, can bind to both RNase E  [ **RNE** ]  and PNPase  [ **PNP** ] , but in contrast to CspE, did not affect the ribonucleolytic actions of these enzymes.  Ref: 11390393 J Biol Chem, 2001
- Earlier work has shown that the C terminal region of RNase E  [ **RNE** ]  is a scaffold for the binding of degradosome components and has identified specific RNase E  [ **RNE** ]  segments necessary for its interaction with polynucleotide phosphorylase  [ **PNP** ]  PNPase  [ **PNP** ] , RhlB RNA helicase, and enolase.  Ref: 11134527 Proc Natl Acad Sci U S A, 2001
- RNase E  [ **RNE** ]  is a large multidomain protein with N terminal ribonucleolytic activity, an RNA binding domain and a C terminal scaffold that binds PNPase  [ **PNP** ] , enolase and RhlB.  Ref: 12035760 Biochem Soc Trans, 2002
- It was already known that an RNase E  [ **RNE** ]  cleavage occurring at the M2 site, ten nucleotides downstream of the coding sequence of rpsO, removes the 3 hairpin which protects the primary transcript from the attack of polynucleotide phosphorylase  [ **PNP** ]  and RNase II.  Ref: 10047480 J Mol Biol, 1999
- However, transiently increasing intracellular poly A levels has also been shown to stabilize the  **pnp**  and  **rne**  transcripts, leading to increased polynucleotide phosphorylase  [ **PNP** ]  PNPase  [ **PNP** ]  and RNase E  [ **RNE** ]  levels respectively.  Ref: 12207699 Mol Microbiol, 2002

**Non-interactive relationship** (e.g. studied together, co-existance, homology, etc.)

- The most important recent developments have been the discovery that the degradosome a multiprotein complex containing an endoribonuclease RNase E  [ **RNE** ] , an exoribonuclease polynucleotide phosphorylase  [ **PNP** ] , and a DEAD box helicase RhlB has a central role in mRNA degradation.  Ref: 10684583 Bioessays, 2000
- Using an E. coli two hybrid system, together with BIAcore apparatus, we investigated the ability of three proteins, polynucleotide phosphorylase  [ **PNP** ]  PNPase  [ **PNP** ] , RhlB RNA helicase, and enolase, a glycolytic protein, to interact physically and functionally independently of RNase E  [ **RNE** ] .  Ref: 12181321 J Biol Chem, 2002
- Furthermore, in the absence of RNase E  [ **RNE** ] , purified RhlB can carry out ATP dependent unwinding of double stranded RNA and consequently modulate degradation of double stranded RNA together with the exonuclease activity of PNPase  [ **PNP** ] .  Ref: 12181321 J Biol Chem, 2002
- Deletion analysis mapped the catalytic domain of RNase ES near its centre and showed that regions located near the RNase ES termini interact with an S. coelicolor homologue of polynucleotide phosphorylase  [ **PNP** ]  PNPase  [ **PNP** ]  a major component of E. coli RNase E  [ **RNE** ]  based degradosomes.  Ref: 12675796 Mol Microbiol, 2003
- It was also found that introduction of an  **rne**  1  **pnp**  7 double mutation restored the expression of mutant lacZ genes with only UCA codons at position 6 or 462.  Ref: 10913980 Can J Microbiol, 2000
- Polynucleotide phosphorylase  [ **PNP** ] , RNase II and RNase E  [ **RNE** ]  play different roles in the in vivo modulation of polyadenylation in Escherichia coli.  Ref: 10844684 Mol Microbiol, 2000
- RNase E  [ **RNE** ]  and PNPase  [ **PNP** ]  are found in a multienzyme complex called the degradosome.  Ref: 10690408 Annu Rev Genet, 1999
- These results provide evidence for the first time that both functional and physical interactions of individual degradosome protein components can occur in the absence of RNase E  [ **RNE** ]  and raise the prospect that the RNase E  [ **RNE** ]  independent complexes of RhlB RNA helicase and PNPase  [ **PNP** ] , detected in vivo, may constitute mini machines that assist in the degradation of duplex RNA in structures physically distinct from multicomponent RNA degradosomes.  Ref: 12181321 J Biol Chem, 2002
- The multifunctional ribonuclease RNase E  [ **RNE** ]  and the 3 exonuclease polynucleotide phosphorylase  [ **PNP** ]  PNPase  [ **PNP** ]  are major components of an Escherichia coli ribonucleolytic machine that has been termed the RNA degradosome.  Ref: 11390393 J Biol Chem, 2001
- In addition, experiments using  **pnp**  lacZ and  **rne**  lacZ translational fusions demonstrate that the variations in transcript stability and protein levels arise from alterations in the autoregulation of both genes.  Ref: 12207699 Mol Microbiol, 2002
- DEAD box RhlB RNA helicase physically associates with exoribonuclease PNPase  [ **PNP** ]  to degrade double stranded RNA independent of the degradosome assembling region of RNase E  [ **RNE** ] .  Ref: 12181321 J Biol Chem, 2002
- Here, we have investigated the role that such nucleases play in modulating polyadenylation in vivo by measuring total poly A levels, polyadenylation of specific transcripts, growth rates and cell viabilities in strains containing various amounts of poly A polymerase I PAP I, polynucleotide phosphorylase  [ **PNP** ]  PNPase  [ **PNP** ] , RNase II and RNase E  [ **RNE** ] .  Ref: 10844684 Mol Microbiol, 2000
- Escherichia coli poly A binding proteins that interact with components of degradosomes or impede RNA decay mediated by polynucleotide phosphorylase  [ **PNP** ]  and RNase E  [ **RNE** ] .  Ref: 11390393 J Biol Chem, 2001
- Our results indicate that RNase E  [ **RNE** ]  associated with the degradosome may contribute to the removal of poly A tails from 5 monophosphorylated RNAs, but this is only likely to be significant should their attack by PNPase  [ **PNP** ]  be blocked.  Ref: 11328869 Nucleic Acids Res, 2001
- The trans acting proteins are mainly four nucleases, two endo RNase E  [ **RNE** ]  and RNase III and two exonucleases PNPase  [ **PNP** ]  and RNase II, and poly A polymerase.  Ref: 10690408 Annu Rev Genet, 1999
- Polyadenylation of Escherichia coli transcripts plays an integral role in regulating intracellular levels of polynucleotide phosphorylase  [ **PNP** ]  and RNase E  [ **RNE** ] .  Ref: 12207699 Mol Microbiol, 2002
- In Escherichia coli, the exoribonuclease polynucleotide phosphorylase  [ **PNP** ]  PNPase  [ **PNP** ] , the endoribonuclease RNase E  [ **RNE** ] , a DEAD RNA helicase and the glycolytic enzyme enolase are associated with a high molecular weight complex, the degradosome.  Ref: 11680851 RNA, 2001
- Our results demonstrate that a degradosome like complex containing RNase E  [ **RNE** ] , PNPase  [ **PNP** ] , and RhlB can form spontaneously in vitro in the absence of all other cellular components.  Ref: 10521403 Genes Dev, 1999
- Thus, it appears that polyadenylation of transcripts in E. coli serves as a sensing mechanism by which the cell adjusts the levels of both RNase E  [ **RNE** ]  and PNPase  [ **PNP** ] .  Ref: 12207699 Mol Microbiol, 2002
- Here, we show that the half lives of both the  **pnp**  and  **rne**  transcripts are dependent on the intracellular level of polyadenylated transcripts.  Ref: 12207699 Mol Microbiol, 2002
- The enzymes polynucleotide phosphorylase  [ **PNP** ] , poly A polymerase and RNase E  [ **RNE** ]  have been demonstrated to have roles in both messenger and RNAI decay.  Ref: 10589716 Microbiology, 1999
- In contrast, the transcripts for  **rne**  RNase E  [ **RNE** ]  and  **pnp**  polynucleotide phosphorylase  [ **PNP** ] , PNPase  [ **PNP** ] , enzymes involved in mRNA decay, were stabilized.  Ref: 10594833 Mol Microbiol, 1999
- Whereas PNPase  [ **PNP** ]  and enolase are present in E. coli in large excess relative to RNase E  [ **RNE** ]  and therefore are detected in cells largely as molecules unlinked to the RNase E  [ **RNE** ]  scaffold, immunogold labeling and biochemical analyses show that helicase is present in approximately equimolar amounts to RNase E  [ **RNE** ]  at all cell growth stages.  Ref: 11134527 Proc Natl Acad Sci U S A, 2001
